# Porphyrien

**DOI:** 10.1007/s00108-021-01066-1

**Published:** 2021-06-29

**Authors:** Ulrich Stölzel, Thomas Stauch, Ilja Kubisch

**Affiliations:** 1 Porphyrie Zentrum, Klinikum Chemnitz gGmbH, Flemmingstr. 2, 09009 Chemnitz, Deutschland; 2 Porphyrie-Speziallabor EPNET, MVZ Labor PD Dr. med. Volkmann und Kollegen GbR, Gerwigstr. 67, 76131 Karlsruhe, Deutschland

**Keywords:** Häm, Bauchschmerz, Lähmungen, Lichtempfindlichkeit, Leberschaden, Heme, Abdominal pain, Paresis, Photosensitivity, Liver damage

## Abstract

Porphyrien werden durch Enzymdefekte der Hämbiosynthese hervorgerufen und anhand spezifischer biochemischer Muster von Porphyrinen und deren Vorläufern in Urin, Stuhl und Blut diagnostiziert. Das jeweilige Muster der akkumulierten Porphyrine, Vorläufer und Derivate ist verbunden mit der klinischen Ausprägung, die abdominale, neurologische, psychiatrische, endokrine, kardiovaskuläre Symptome, Leberschaden und/oder Lichtempfindlichkeit der Haut umfassen kann. Klinisch werden akute und nichtakute Porphyrien unterschieden. Bei symptomatischen (klinisch aktiven), akuten hepatischen Porphyrien – hierzu gehören akute intermittierende Porphyrie, Porphyria variegata, hereditäre Koproporphyrie und Doss-Porphyrie – kommt es aufgrund einer Regulationsstörung zur Kumulation der Porphyrinvorläufer 5‑Aminolävulinsäure und Porphobilinogen. Bei den nichtakuten Formen – u. a. Porphyria cutanea tarda, erythropoetische und X‑chromosomale Protoporphyrie sowie kongenitale erythropoetische Porphyrie – führen akkumulierte Porphyrine zu Lichtempfindlichkeit (Fotodermatose) und mitunter auch zu schweren Leberschäden. Zur Therapie der Porphyrien stehen sowohl bewährte und sichere als auch innovative Optionen zur Verfügung.

## Abstract (en)

Porphyrias are caused by enzyme defects along the heme biosynthetic pathway. The first line diagnosis of porphyria is based on specific biochemical patterns of elevated porphyrins and porphyrin precursors in urine, feces, and blood. In clinically active disease accumulated porphyrin precursors and/or porphyrins lead to abdominal, neurologic, psychiatric, endocrine and cardiovascular symptoms, liver damage and/or skin photosensitivity. Porphyrias are classified into acute and nonacute forms. Patients with symptomatic (clinically active) acute hepatic porphyria, e.g. acute intermittent porphyria, porphyria variegata, hereditary coproporphyria, and aminolevulinic acid dehydratase deficiency porphyria, display accumulation of porphyrin precursors, 5‑aminolevulinic acid and porphobilinogen due to regulation disorder. In the non-acute forms of porphyria, such as porphyria cutanea tarda, erythropoietic porphyria, X‑linked protoporphyria and congenital erythropoietic porphyria, accumulated porphyrins lead to skin photosensitivity and occasionally also to severe liver damage. Several different options for treatment, proven and innovative ones, are available for most porphyrias.

## Lernziele

Nach Lektüre dieses Beitrags

- werden Sie Porphyrien in die differenzialdiagnostischen Überlegungen einbeziehen.
- werden Sie über Symptome und Diagnose von Porphyrien klare Vorstellungen entwickeln.
- werden Sie neue Therapieprinzipien kennenlernen.
- werden Sie häufigen Fehlern und Fallstricken ausweichen können.
- werden Sie Ihr allgemeininternistisches Wissen fachübergreifend schulen.

## Einleitung

Häm ist funktioneller Bestandteil des Hämoglobins, Myoglobins, von Enzymen der Zytochrom-P450-Familie, der mitochondrialen Zytochrome und weiterer Enzyme. Es wird überwiegend im Knochenmark (80 %) und in der Leber (20 %) synthetisiert. Porphyrien sind Störungen der **Hämbiosynthese**

**Figure 1:**
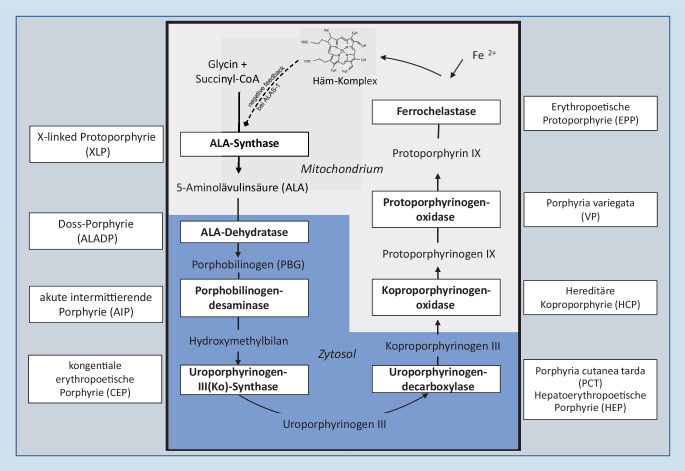

Hämbiosynthese

([1]; Abb. 1). Sie werden klinisch in *akute und nichtakute Porphyrien* sowie entsprechend dem Hauptmanifestationsorgan der Synthesestörung in *hepatische bzw. erythropoetische Porphyrien* differenziert.

Im vorliegenden CME-Beitrag werden eine Kurzkasuistik, ferner die unterschiedlichen klinischen Präsentationen, Diagnostiken und Therapien der Porphyrien behandelt (Tab. 1). In welchem Gewebe sich eine Hämsynthesestörung a priori auswirkt, wird durch Art und Ausmaß des jeweiligen **Enzymdefekts**

Enzymdefekts

und die Regulation der Hämsynthese in den betreffenden Zellen bestimmt. So kommt es z. B. bei Flaschenhalsdefekten (Störungen, die leistungsbegrenzende Schritte der Hämsynthese betreffen), gepaart mit einem Feedbackinhibitionsmechanismus durch Häm als Endprodukt, zur Ausprägung der akuten hepatischen Porphyrien. Die Feedbackhemmung des Initialschrittes der Hämsynthese, der in der Bildung der **5‑Aminolävulinsäure**

5‑Aminolävulinsäure

besteht, ist Grundlage der pulsatilen Hämbereitstellung in der Leber (z. B. nach Zufuhr von Xenobiotika, nach verstärkter Hämdegradation bei Akute-Phase-Reaktion etc.) und gewährleistet eine rasche Adaptation an exogene oder endogene Änderungen des hepatischen Hämbedarfs. Dieser (negative) Steuerungsmechanismus ist im Gegenzug naturgemäß anfällig für metabolische Dysregulationen im Sinne sog. **Regulationskrankheiten**

Regulationskrankheiten

[2].

**Table 1:** 

Porphyrien und Bleivergiftung	Enzymaktivität	Diagnostische Charakteristika		Plasmascreen (nm)^h^	Neuroviszerale Symptome	Kutane Symptome	Anämie	Leberschaden
		„First line“	„Second line“					
*Akute hepatische Porphyrien*								
Akute intermittierende Porphyrie (AIP)	PBGD ↓	Im Urin ALA ↑↑, PBG ↑↑ und Porphyrine ↑↑	Gensequenzierung PBGD^f^	618–620	++	−	−	±^j^
Porphyria variegata (VP)	PPOX↓	Im Urin ALA ↑↑, PBG ↑↑ und Porphyrine ↑↑	Gensequenzierung fäkales Koproporphyrin ↑ und PPXI ↑	625–627	+	±	−	±
Hereditäre Koproporphyrie (HCP)	CPOX^a^↓	Im Urin ALA ↑↑, PBG ↑↑ und Porphyrine ↑↑	Gensequenzierung fäkales Koproporphyrin ↑	618–620	+	±	−	±
5‑Aminolävulinsäure-Dehydratase-Defekt Porphyrie (ALADP)	ALAD ↓	Im Urin ALA ↑↑, PBG normal oder ↑	Gensequenzierung	615–619	++	−	±	−
*Weitere (nichtakute) hepatische Porphyrien*								
Porphyria cutanea tarda (PCT)	– UROD↓	Im Urin Porphyrine ↑↑	Gensequenzierung UROD^g^	618–620	−	+	−	+
Hepatoerythropoetische Porphyrie (HEP)		Uro- > > Koproporphyrin^c^			−	++	+	+
*Erythropoetische Porphyrien*								
Erythropoetische Protoporphyrie (EPP)	FECH↓	In Erythrozyten freies-PPIX ↑↑, Zink-PPIX^d ↑^	Gensequenzierung	634–635	−	++	±	±
X‑linked Protoporphyrie (XLP)	ALAS2^b^↑	In Erythrozyten freies-PPIX ↑↑, Zink-PPIX^e^ ↑↑	Gensequenzierung	624–635	−	++	±	±
Kongenitale erythropoetische Porphyrie (CEP)	UROS ↓	In Urin und Stuhl Uro- und Koproporphyrin isomer I ↑↑	Gensequenzierung	615–618	−	++	+	−
*Andere*								
Bleivergiftung	ALAD ↓	Im Urin ALA ↑↑, PBG normal oder ↑	Im Blut: Bleikonzentration ↑	615–618	++	−	+^i^	+

Reihenfolge geordnet nach Kategorie und Prävalenz

Abkürzungen s. Abkürzungsverzeichnis

*↑* erhöht, *↓* erniedrigt, *−, ±, +* klinische Zeichen nicht, variabel oder typisch präsent bei klinischer Manifestation

^a^Eine spezifische homozygote Mutation oder ein Nullallel im *CPOX*-Gen löst die seltene Harder-Porphyrie aus, die (phänotypisch unterschiedlich) nicht zu abdominalen und neurologischen Symptomen führt

^b^5‑Aminolävulinsäure-Synthase‑2 im Knochenmark

^c^↑ fäkales Isokoproporphyrin ist ein spezifischer Marker für die PCT/HEP, nur bei HEP werden erhöhte Werte des freien und zinkgebundenen Protoporphyrins in Erythrozyten gefunden

^d^Bei EPP ist der Anteil an Zink gebundenem vs. freiem Protoporphyrin signifikant geringer (< 15 %) im Vergleich zur XLP

^e^Bei XLP ist der Anteil an zinkgebundenem vs. freiem Protoporphyrin > 25 %

^f^↓ Enzymaktivität im Blut, normal nur in der nichterythroiden Spleißmutationsvariante

^g^↓ Enzymaktivität im Blut, normale Aktivität im Blut nur in der erworbenen Form der PCT (Typ 1)

^h^Fluoreszenzemissionsmaximum (nm) von Plasmaporphyrinen bei Exzitation mit 405 nm

^i^Mikrozytäre Anämie bei Bleivergiftung, da Blei die Ferrochelatase hemmt

^j^Konzentrationserhöhungen von ALT und AST bis zum 3‑Fachen der Norm in der Akutphase, erhöhtes Risiko für ein HCC

Störungen im Bereich zunächst nichtleistungsbegrenzender Passagen der Hämbiosynthese gemeinsam mit einer stimulativen (positiven) Regulation, z. B. Eisen im Rahmen der Erythropoese, führen hingegen lediglich zu einer Akkumulation entsprechender Intermediate, die der beeinträchtigten Umsetzung vorausgehen. Da hierbei keine Dysregulation besteht bzw. entsteht, spricht man von den sog. **Speicherkrankheiten**

Speicherkrankheiten.

Die „Speicherung“ der anflutenden Intermediate ist ursächlich für das resultierende klinische Beschwerdebild bzw. die mit diesen Störungen assoziierten Symptome.

**Figure 2:**
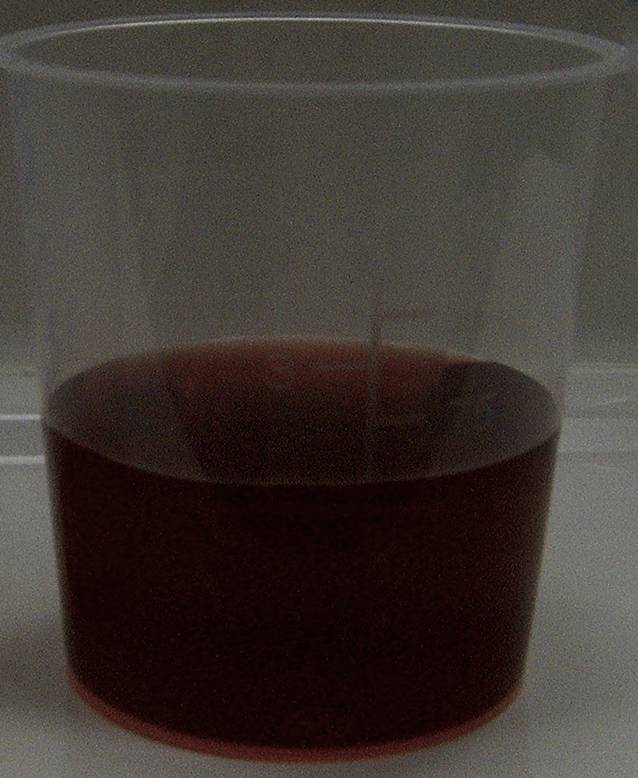

### Fallbeispiel.

Eine 28-jährige Frau klagte über zyklusabhängige Bauchschmerzen. Eine umfangreiche Diagnostik ergab keinen wegweisenden Befund. Eine psychosomatische Behandlung wurde empfohlen. Im Verlauf verstärkten sich die Beschwerden nach einer Fastenkur. Es traten schmerzhafte Parästhesien in den Oberschenkeln, Übelkeit, Obstipation und Erbrechen auf. Symptomatisch wurde Metamizol verordnet. Beginnende Lähmungen der Streckmuskulatur beider Hände, eine schwere Hyponatriämie (Natriumkonzentration 105 mmol/l) und Halluzinationen führten zu stationärer Aufnahme und sofortiger Intensivtherapie. Nachdem im Urinbeutel rötlicher Urin (Abb. 2) und in einer Urinprobe massiv erhöhte ALA- und PBG-Konzentrationen gefunden wurden, konnte die Diagnose einer AHP gestellt werden. Es wurde mit Hämarginat i.v. (3 mg/kgKG in 100 ml 20 %iger Albuminlösung über 30 min) für 4 Tage therapiert. Wegen des Erbrechens musste die kalorische Versorgung parenteral über Infusionen erfolgen. In der Akutphase wurde eine Kalorienmenge von 24 kcal/kgKG und Tag angestrebt. Im Hinblick auf die individuelle metabolische Toleranz erfolgten tägliche Serumphosphatbestimmungen sowie 6‑stündlich Blutzuckerkontrollen zur Vermeidung eines Refeeding-Syndroms.

Die Hyponatriämie wurde mithilfe einer Infusion von 150 ml einer hyperosmolaren Natriumchloridlösung (3 %ig) über 20 min therapiert. Nachfolgend wurde die Serumnatriumkonzentration kontrolliert. Dieser Therapieschritt wurde wiederholt, bis ein Anstieg um 5 mmol/l erreicht war. Die weitere Infusionstherapie erfolgte mit isotonische Natriumchloridlösung (0,9 %) unter initial stündlicher Überwachung der Serumnatriumkonzentration. Es wurde beachtet, den Serumnatriumwert in den ersten 24 h nicht über 10 mmol/l zu erhöhen, in den folgenden Tagen nicht über 8 mmol/l und Tag. Unter diesem Therapieregime erholte sich die Patientin rasch; die Lähmungen bildeten sich zurück. Die Ernährung war wieder enteral möglich.

## Genetik

Porphyrien zeichnen sich durch eine **molekulargenetische Heterogenität**

molekulargenetische Heterogenität

aus (Tab. 2). Die bislang identifizierten Mutationen sind u. a. in der Human Gene Mutation Database (www.hgmd.cf.ac.uk) erfasst. Mit Ausnahme von ALADP, der HEP und CEP, die autosomal-rezessiv vererbt werden, sowie der ALAS2-abhängigen Protoporphyrie XLP mit X‑chromosomalem Erbgang werden hereditäre Porphyrien autosomal-dominant vererbt. Die Mutation auf einem Allel führt häufig zu einer Halbierung der Enzymaktivität, wobei diese Mangelzustände oft durch eine großzügige Auslegung der jeweiligen Umwandlungskapazitäten kompensiert werden und sich lediglich unter ungünstigen Umständen bei zusätzlichen Einschränkungen und/oder extremen Hämabforderungen z. B. unter exogen-toxischen Einflüssen auswirken (geringe phänotypische Penetranz). Bei der EPP wird auf dem nicht von der Hauptmutation betroffenen zweiten Allel ein Polymorphismus gefunden, der die phänotypische Expression bedingt, ein so genannter **Mindersynthesepolymorphismus**

Mindersynthesepolymorphismus

oder eine hypomorphe Mutation. Sind beide gegenüberliegenden Allele mit funktioneller Auswirkung unterschiedlich verändert/mutiert, liegt eine **Compound-Heterozygotie**

Compound-Heterozygotie

vor (z. B. im Fall einer ALADP oder CEP). Bei ansonsten autosomal-dominant vererbten, akuten hepatischen Porphyrien finden sich selten auch homozygote bzw. compound-heterozygote Formen.

**Table 2:** 

Porphyrie	OMIM-Nr.	Anzahl der bisher bekannten Mutationen	Genloci	Publizierte Prävalenz^a^	Diagnostizierte Porphyrien^b^
ALADP	612740	13	9q32	Selten^c^	3
AIP	176000	421	11q23.2	10	909
CEP	263700	51	10q26.2	Selten	35
PCT, HEP^d^	176100	122	1p34.1	40	3175
HCP	121300	71	3q12.1	1	82
VP^e^	176200	184	1q23.3	3	144
EPP	177000	195	18q21.31	9	302
XLP	300752	4	Xp11.2	Selten^f^	3

Abkürzungen s. Abkürzungsverzeichnis

^a^Prävalenz der Erkrankung: Fälle/1.000.000 Einwohner

^b^Porphyrien, die im „German Competence Center for Porphyria Diagnostics and Consultation“ von 1965 bis 2019 gefunden wurden: 4550 Porphyrien in folgender Proportion PCT: AIP: EPP: VP: HCP: CEP = 91: 26: 9: 4: 2: 1

^c^Bisher 6 Fälle beschrieben

^d^Bisher 20 Fälle mit HEP beschrieben

^e^Hohe Prävalenz in Südafrika und Südamerika (Founder-Effekt)

^f^Seit der Erstbeschreibung der XLP 2008 konnten etwa 2–10 % der EPP der XLP nachträglich zugeordnet werden

## Epidemiologie

Die klinisch häufigsten Formen sind Porphyria cutanea tarda (PCT), akute intermittierende Porphyrie (AIP) und erythropoetische Protoporphyrie (EPP). Zwischen 1965 und 2017 wurden im „German Competence Center for Porphyria Diagnosis and Consultation“ mehr als 4500 Porphyrien diagnostiziert (Tab. 2).

### Merke

Die klinisch häufigsten Formen sind Porphyria cutanea tarda (PCT), akute intermittierende Porphyrie (AIP) und erythropoetische Protoporphyrie (EPP).

## Pathophysiologie

### Akute hepatische Porphyrien (AIP, VP, HCP, ALADP)

Akuten hepatischen Porphyrien liegen – genetisch bedingt – defekte Enzyme der Hämsynthese zugrunde (Tab. 1). Erst die Induktion der **hepatischen Hämsynthese**

hepatischen Hämsynthese

durchbricht die Latenz und löst klinische Symptome aus. Porphyrinogene Arzneistoffe (www.drugs-porphyria.org), Xenobiotika, Sexualhormone, Alkohol, Nikotin, Nahrungskarenz oder erhöhter Energieverbrauch, z. B. durch Ausdauersport o. Ä., erzeugen einen erhöhten Hämbedarf und stimulieren die hepatische Hämsynthese. Die unmittelbare Interaktion mit definierten Sequenzen des *ALAS1*-Gens („drug-responsive elements“) und nukleären Rezeptoren (z. B. CAR, PXR) vermitteln die hierfür erforderliche Induktion der **hepatischen 5‑Aminolävulinsäure-Synthase 1**

hepatischen 5‑Aminolävulinsäure-Synthase 1

(ALAS1) auf molekularer Ebene. Darüber hinaus führen Stress, Entzündung und Infektionen zur Induktion der **Hämoxygenase‑1**

Hämoxygenase‑1,

einem Akute-Phase-Reaktanden [3]. Über dieses Enzym wird Häm abgebaut. Kompensatorisch kommt es zu erhöhter Synthese von Porphyrinvorläufern (ALA und PBG) sowie Porphyrinen [4].

Durch den Konzentrationsanstieg des **Porphobilinogens**

Porphobilinogens

und damit ein erhöhtes Substratangebot zur Bildung von Uroporphyrin wird der partielle Enzymmangel bei der AIP größtenteils kompensiert. Trotz genetisch verminderter Enzymaktivität kommt es zu hinreichender Porphyrinogensynthese, um den hepatischen Hämbedarf zu sichern. Dieses wichtige Phänomen erklärt dynamisch nicht nur erhöhte Konzentrationen von ALA und PBG im Urin, sondern auch von Porphyrinen. Die zunächst paradox erscheinende Erhöhung von Metaboliten „stromabwärts“ des Enzymdefekts veranschaulicht das „Durchbrechen“ des pathophysiologischen Engpasses im Zuge der Hochregulation der Porphyrinvorläufer und illustriert, warum die akuten Porphyrien als Dysregulationskrankheiten zu verstehen sind. Die erhöhten Konzentrationen der Porphyrine bedingen die **Urinrotfärbung**

Urinrotfärbung.

Der exkretorische Anstieg von ALA und PBG bei HCP und VP resultiert, neben den genannten regulatorischen Gründen, u. a. aus der Hemmung der **hepatischen Porphobilinogendesaminase**

hepatischen Porphobilinogendesaminase

durch akkumulierende Metabolite.

#### Merke

Zwei Mechanismen führen zu erhöhter Synthese der Porphyrinvorläufer ALA und PBG sowie von Porphyrinen bei akuten hepatischen Porphyrien:

1. die Induktion der hepatischen ALAS1 (direkt),
2. die reduzierte oder fehlende Feedbackhemmung der Exprimierung des Enzyms durch das Endprodukt Häm (indirekt).

### Porphyria cutanea tarda (PCT, HEP)

Es gibt eine erworbene (Typ 1) und eine familiäre Form (Typ 2)[5]. Bei der **familiären Form**

familiären Form

bewirken heterozygote Mutationen im *UROD*-Gen eine Aktivitätsminderung um etwa 50 %. Meist führen zusätzliche Trigger zum Ausbruch der Erkrankung (Tab. 3). Ein wichtiger Prädispositionsfaktor ist **Eisen**

Eisen,

das Uroporphyrinogen zu Uroporphomethen, einem kompetitiven Inhibitor der UROD, oxidiert [6]. Bei etwa zwei Dritteln der Patienten werden *HFE*(Hereditary Fe Gen)-Gen-Mutationen gefunden [7]. Die PCT kann ferner durch Hexachlorbenzol, Dioxin, Alkohol und hormonale Kontrazeptiva/postmenopausale Hormonersatztherapie ausgelöst werden [8]. Bei chronischer Niereninsuffizienz mit Hämodialyse werden gelegentlich ähnliche Hautveränderungen gefunden, die als **Pseudoporphyrie**

Pseudoporphyrie

bezeichnet werden. Die Differenzialdiagnose Pseudoporphyrie vs. echte PCT ist eine der seltenen Indikationen für eine **Porphyrinbestimmung**

Porphyrinbestimmung

im Serum bzw. im Plasma. Mit der PCT sind gehäuft Hepatitis-C- und auch HIV-Infektionen assoziiert [9].

**Table 3:** 

Erworbene Faktoren	Genetische Faktoren
Lebersiderose	*UROD*-Mutationen
Hexachlorbenzol	*HFE*-Mutationen
Alkohol	D519G-Polymorphismen des *GNPAT*-Gens^b^
Östrogene	*CYP*-Gene^c^
Hepatitis-C- und HIV-Infektion	–

^a^Erworbene und genetische Faktoren führen zu einer verminderten (< 20 %) Aktivität der UROD in der Leber und zur klinischen Manifestation der PCT

^b^Patienten mit familiärer PCT (Typ 2) exprimieren häufiger *GNPAT*-Gen-Polymorphismen, die Hepcidin negativ regulieren und zur Eisenakkumulation bei HFE-assoziierter Hämochromatose beitragen

^c^Patienten mit erworbener PCT (Typ 1) weisen häufig *CYP*-Gen-Polymorphismen, die zu erhöhter Enzymaktivität prädisponieren, auf

Die **hepatoerythropoetische Porphyrie**

hepatoerythropoetische Porphyrie

(HEP) ist eine seltene homozygote oder compound-heterozygote Form, mit ausgeprägt erniedrigter UROD-Aktivität (< 20 %). Die Manifestation der **Fotodermatose**

Fotodermatose

und einer milden hämolytischen Anämie erfolgt bereits in der Kindheit.

#### Merke

Ein wichtiger Prädispositionsfaktor ist hepatisch akkumuliertes Eisen.

### Erythropoetische Protoporphyrien

Aufgrund eines Defekts der **Ferrochelatase**

Ferrochelatase

steigt die Protoporphyrinkonzentration in den Erythrozyten (> 4500 nmol/l, normal < 89 nmol/l). Es handelt sich überwiegend um freies Protoporphyrin im Gegensatz zum zinkgebundenen Protoporphyrin bei sekundären Protoporphyrinämien (z. B. bei Eisenmangel und Bleiintoxikation). Das lipophile Protoporphyrin wird biliär eliminiert und ist in höheren Konzentrationen toxisch. Histologisch können protoporphyrinhaltige Strukturen als **„Malteserkreuze“**

**Figure 3:**
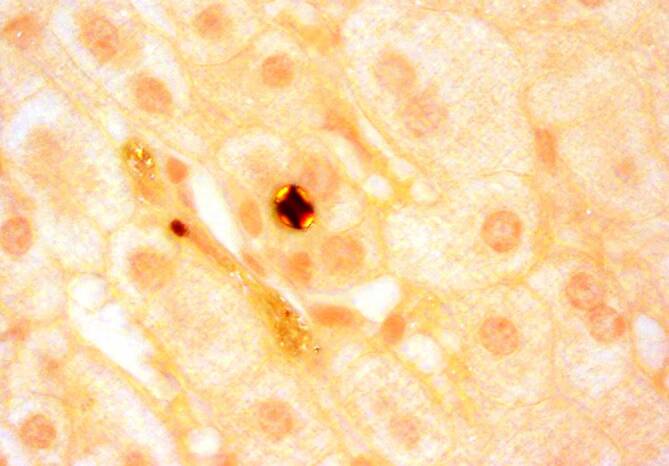

„Malteserkreuze“

im Lebergewebe nachgewiesen werden (Abb. 3). Diese sind für die EPP und XLP pathognomonisch. **Protoporphyrinhaltige Gallensteine**

Protoporphyrinhaltige Gallensteine,

die unter langwelligem UV-Licht rot fluoreszieren, kommen bei etwa 10 % der Patienten vor. Im Jahr 2008 wurde eine neu gefundene X‑chromosomal vererbte Deletion im *ALAS2*-Gen publiziert (betrifft den ersten Schritt der erythropoetischen Hämbiosynthese, [10]). Diese **hypermorphen Mutationen**

hypermorphen Mutationen

führen zu einer neuen Protoporphyrie (XLP) mit erhöhter ALAS2-Enzym-Konzentration („gain-of-function mutation“) und sich daraus ergebender Akkumulation von freiem, aber auch zinkgebundenem Protoporphyrin.

#### Merke

Bei Protoporphyrien kommt es zu einer massiven Erhöhung von freiem Protoporphyrin im Blut.

### Erythropoetische Harder-Porphyrie

Bei dieser sehr seltenen Erkrankung stagniert die durch die Koproporphyrinogenoxidase katalysierte Umwandlung von Koproporphyrin in Protoporphyrin nach einem Decarboxylierungssschritt. Folglich akkumuliert **tricarboxyliertes Porphyrin**

tricarboxyliertes Porphyrin

(Harder-Porphyrin) in Erythrozyten und wird vermehrt im Stuhl ausgeschieden. Ursache ist eine spezifische homozygote Mutation oder ein Nullallel im Exon 6 der Koproporphyrinogenoxidase.

### Kongenitale erythropoetische Porphyrie

Die kongenitale erythropoetische Porphyrie (CEP) oder **M. Günther**

M. Günther

wurde nach dem Leipziger Arzt Hans Günther benannt, der diese seltene angeborene Erkrankung 1911 beschrieben hat. Die verfügbare Aktivität bzw. Restmenge des Enzyms **„Uroporphyrinogen-III-Kosynthase“**

„Uroporphyrinogen-III-Kosynthase“

(UROS) ist aufgrund von compound-heterozygoten oder homozygoten Genmutation, die die Stabilität des Enzymproteins vermindern und zu dessen rascher Degradation führen, sehr gering. Dies führt infolge spontaner (nichtenzymatischer) Zyklisierung von Hydroxymethylbilan zu Uroporphyrinogen I und damit zu einer Anflutung von Porphyrinogenen der Isomerenreihe I, deren weitere Metabolisierung beim Koproporphyrinogen I in einer Sackgasse endet, da die weitere Umsetzung zu Häm nicht möglich ist. Die Porphyrinogene oxidieren anteilig spontan zu Porphyrinen und akkumulieren ubiquitär (z. B. fluoreszierende Zähne).

## Klinische Zeichen

### Akute hepatische Porphyrien (AIP, VP, HCP, ALADP)

Akute hepatische Porphyrien manifestieren sich meist nicht vor der Pubertät. Frauen sind häufiger betroffen als Männer (3–4:1).

**Abdominalschmerzen**

**Figure 4:**
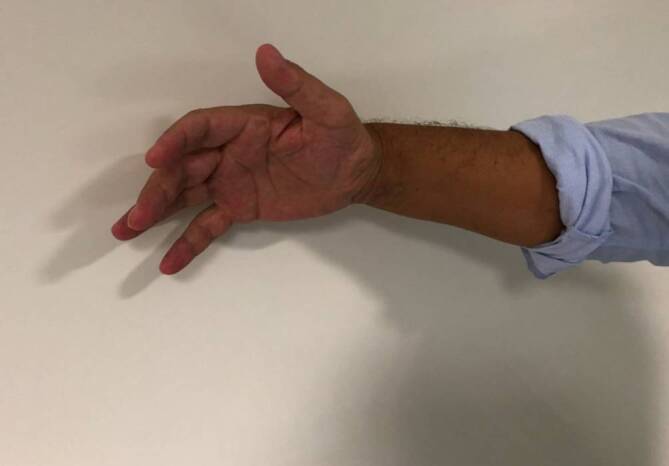

Abdominalschmerzen,

intermittierend und kolikartig, sind ein initiales und häufiges Symptom, gleichzeitig oder später evtl. Rücken‑, Extremitätenschmerzen und Parästhesien. Den Schmerzen im mittleren und im unteren Abdomen folgen evtl. Obstipation, Übelkeit, Erbrechen und eine Ileussymptomatik. Tachykardie, Hypertonie und ein rot nachdunkelnder Urin (Abb. 2) sind wichtige diagnostische Hinweise. Bei Nichterkennen, Fortschreiten oder Verstärkung des Porphyrieprozesses durch inadäquate Maßnahmen (Medikamente, Fehlernährung etc.) und Fehldiagnosen kommt es zur **peripheren motorischen Neuropathie**

peripheren motorischen Neuropathie,

die zuerst die Streckmuskulatur an Händen (Abb. 4) und Armen befällt. Die Lähmungen können aufsteigen und eine Tetraparese mit Atemlähmung auslösen. Bei einigen Patienten kommt es zu Vigilanzstörungen, Krampfanfällen, Verstimmungs- und/oder Erregungszuständen bis hin zu Halluzinationen. Es gibt Hinweise, dass die Prävalenz akuter Porphyrien bei psychiatrisch Kranken deutlich höher ist. Eine inadäquat hohe ADH-Sekretion (**Schwartz-Bartter-Syndrom**

Schwartz-Bartter-Syndrom)

geht mit einer Hyponatriämie einher, die bei abdomineller Symptomatik für die Diagnose eines akuten hepatischen Porphyriesyndroms wegweisend sein kann. Das Risiko für hepatozelluläre Karzinome sowie hypertensive Nierenschäden ist erhöht.

Bei hereditärer Koproporphyrie und Porphyria variegata können zudem lichtabhängige Hautsymptome auftreten. Bei Frauen kommt es nicht selten zu **prämenstruellen Manifestationen**

prämenstruellen Manifestationen.

Darüber hinaus gibt es inter- und individuell eine erhebliche Variabilität der Polysymptomatik klinischer Manifestationen. Etwa 8 % aller Patienten mit akuten Porphyrien entwickeln rekurrierend klinische Manifestationen und auch chronische Symptome. In der EXPLORE-Studie wurde dies prospektiv und multizentrisch erfasst [11]. Nicht selten haben die Patienten täglich Beschwerden und eine geminderte Lebensqualität.

### Porphyria cutanea tarda (PCT, HEP)

Bei der Porphyria cutanea tarda entstehen **Blasen**

**Figure 5:**
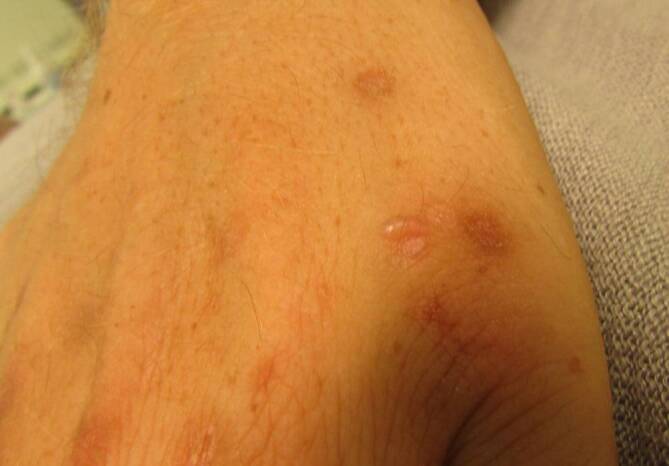

Blasen

(Abb.5) und Narben an sonnenlichtexponierten Hautpartien, leichte Verletzbarkeit an den Händen und Hypertrichose im Schläfen- und im Jochbeinbereich sowie periorbital. Bereits ab einer Urinausscheidung von 2 mmol/Tag ist die Leber voll beladen mit Porphyrinen, sodass der Biopsiezylinder unter langwelligem UV-Licht (366 nm) eine leuchtende intensive **Rotfluoreszenz**

**Figure 6:**
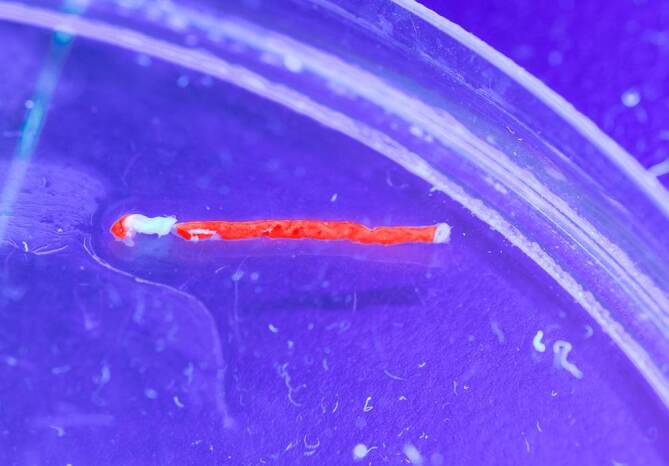

**Figure 7:**
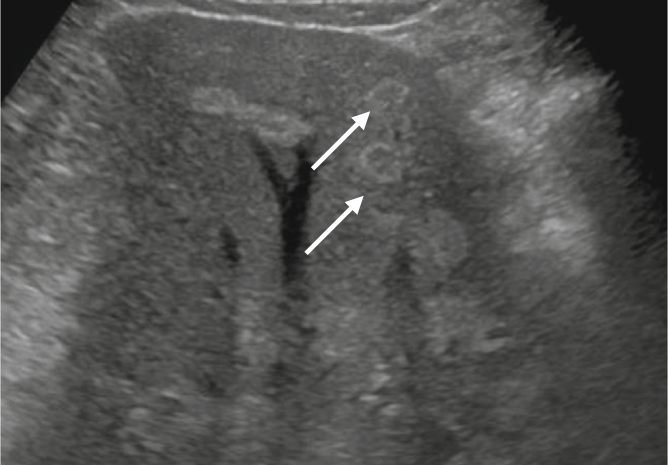

Rotfluoreszenz

zeigt (Abb. 6). Gelegentlich werden bei Porphyria cutanea tarda fokale echoreiche bzw. echoreich ringförmige Leberveränderungen im Ultraschall gefunden, die sich in der Kontrastmittelsonographie nicht vom umgebenden Lebergewebe demarkieren (Abb. 7).

Im Rahmen der Abklärung erhöhter Konzentrationen der Serumaminotransferasen (ALT, AST) können latente PCT-Phasen ohne Hautsymptome erkannt werden.

#### Merke

Im Rahmen der Abklärung erhöhter Konzentrationen der Serumaminotransferasen können latente PCT-Phasen ohne Hautsymptome mithilfe von Sonographie und Analysen der Porphyrine im Urin erkannt werden.

### Protoporphyrien (EPP, XLP)

Die Lichtdermatose bei Protoporphyrie tritt meist schon in der Kindheit auf und geht an den exponierten Hautarealen mit Brennen, Jucken, Schmerzen, Erythemen und Ödemen (**„Sonnenurtikaria“**

„Sonnenurtikaria“)

einher. Dieser erythropoetischen, kutanen Phase folgt bei 25 % der Betroffenen eine **erythrohepatobiliäre Phase**

erythrohepatobiliäre Phase,

die bei ca. 10 % der Protoporphyriepatienten zu Leberzirrhose mit Hepatomegalie, Cholestase, Ikterus und abdominalen Schmerzen führt. Aus klinischer Sicht sollte an die Protoporphyrie bei ungeklärter Cholestase, bei Hepatomegalie und/oder abdominalen Schmerzen gedacht werden.

### Kongenitale erythropoetische Porphyrie

Dominierend ist eine **schwere Fotodermatose**

schwere Fotodermatose

mit Mutilationen bereits im Säuglings- und Kindesalter. Eine Anämie und Splenomegalie entwickeln sich erst später. Porphyrinakkumulation in den Zähnen, die rötlichbraun verfärbt sein können, resultiert in einer **„Erythrodontie“**

„Erythrodontie“;

die Zähne fluoreszieren im langwelligen UV-Licht leuchtend rot. Es gibt Hinweise, dass die Patienten mit CEP von einer **Eisendepletion**

Eisendepletion

profitieren [12]. Im Hinblick auf Diagnostik und Therapie dieser seltenen Erkrankung wird auf weiterführende Literatur verwiesen.

## Diagnostik

### Akute hepatische Porphyrien

Die klinischen Zeichen akuter hepatischer Porphyrien gehen immer mit erheblich erhöhter Ausscheidung beider Porphyrinvorläufer ALA und PBG sowie der Porphyrine im Urin einher, die in der Remissionsphase abfällt und in den Latenzphasen meist noch signifikant über dem jeweiligen Cut-off-Wert liegt. Die hohe ALA- und PBG-Ausscheidung beruht neben der metabolischen Dysregulation infolge des Hämmangels bei AIP auf dem PBGD-Defekt. Diese Enzymreaktion ist physiologisch der sekundär limitierende Schritt („Flaschenhals“) der Porphyrinbiosynthese und führt somit bei HCP und VP zu einer AIP-analogen Exkretionskonstellation. Hohe Metabolitenspiegel ohne klinische Symptome reflektieren eine dekompensierte Latenzphase. Die Betroffenen werden als **symptomfreie Hochausscheider**

symptomfreie Hochausscheider

bezeichnet.

#### Merke

Die klinischen Zeichen akuter hepatischer Porphyrien gehen bei unbehandelten Patienten immer mit einer ausgeprägten erhöhten Ausscheidung beider Porphyrinvorläufer (ALA und PBG) sowie der Porphyrine im Urin einher.

**Metabolitenuntersuchungen**

Metabolitenuntersuchungen

des Porphyrinstoffwechsels in Urin und Stuhl sind obligat (Tab. 3). Anhand der Metabolitenausscheidung können die metabolische und klinische Aktivität sowie die Wirksamkeit der Therapie überprüft werden. Möglicherweise korreliert die zeitliche Dynamik/Steilheit der Exkretionsanstiege (Δc_Vorläufer_/Δt) besser mit der klinischen Situation und der Symptomausprägung als die absolute Wertelage. Auch in beschwerdefreien Phasen wird eine jährliche **Urinuntersuchung**

Urinuntersuchung

empfohlen. Der Urin sollte lichtgeschützt (dunkles Röhrchen oder Umwicklung mit Alufolie) transportiert werden. Eine **Kreatininkonzentrationsmessung**

Kreatininkonzentrationsmessung

im Spontanurin (20 ml) ist ausreichend. Liegt eine mithilfe der Urinanalyse gesicherte akute Porphyrie vor, sollte eine **Stuhluntersuchung**

Stuhluntersuchung

auf Porphyrine zur differenzialdiagnostischen Abgrenzung der VP bzw. der HCP erfolgen. Die Diagnose einer AIP kann durch eine Aktivitätsbestimmung der PBGD und evtl. eine **molekulargenetische Analyse**

molekulargenetische Analyse

bestätigt werden. Da bei biochemisch gesicherter HCP und VP routinetaugliche Enzymtests nicht zur Verfügung stehen, ist ggf. eine molekulargenetische Analyse empfehlenswert. Bei Kenntnis der Mutation des Indexpatienten kann erstgradig Verwandten eine gezielte Genanalyse angeboten werden, um symptomfreie Mutationsträger zu identifizieren und über porphyrieauslösende Situationen zu informieren (Medikamente, Alkohol, Fasten, Rauchen). Bis > 90 % der Genträger (in betroffenen Familien bis 77 %) bleiben lebenslang symptomfrei.

### Porphyria cutanea tarda (PCT, HEP)

Bei der Porphyria cutanea tarda und der sehr seltenen HEP sind die Konzentrationen der Porphyrine in Urin und Plasma extrem erhöht. Es dominieren die höhercarboxylierten Fraktionen Uro- und Heptacarboxyporphyrin. Der Nachweis von **Isokoproporphyrin**

Isokoproporphyrin

im Stuhl ist ein pathognomonischer Befund. Bei der HEP zeigen auch die Konzentrationen der **Erythrozytenporphyrine**

Erythrozytenporphyrine

signifikante Anstiege.

#### Merke

Bei der Porphyria cutanea tarda und der sehr seltenen HEP sind die Porphyrine im Urin und Plasma extrem erhöht. Es dominieren Uro- und Heptacarboxyporphyrin.

### Erythropoetische Protoporphyrie

Die Protoporphyrie wird durch erhöhte Konzentrationen von freiem Protoporphyrin (> 4500 nmol/l, Referenzbereich < 89 nmol/l) im antikoagulierten Vollblut (Erythrozyten und Plasma) diagnostiziert. Bei XLP ist zusätzlich der Anteil des zinkgebundene Protoporphyrins (> 25 %) erhöht. Im Verlauf ansteigende erythrozytäre Protoporphyrinkonzentrationen (Gesamtprotoporphyrin > 25.000 nmol/l; Referenzbereich < 500 nmol/l) und eine Dominanz von **Koproporphyrinisomer I**

Koproporphyrinisomer I

gegenüber Koproporphyrinisomer III im Urin können prognostisch die Gefahr einer Leberbeteiligung anzeigen.

#### Merke

Die Protoporphyrie wird durch erhöhte Konzentrationen von freiem Protoporphyrin im antikoagulierten Vollblut (Erythrozyten und Plasma) diagnostiziert. Bei XLP ist zusätzlich das zinkgebundene Protoporphyrin IX erhöht.

### Erythropoetische Harder-Porphyrie

Diagnostisch wegweisend ist die erhöhte fäkale Exkretion von tricarboxyliertem Porphyrin (Harder-Porphyrin). Mutationsanalysen im *CPOX*-Gen bestätigen die Diagnose.

### Differenzialdiagnose

Erhöhte Porphyrinausscheidungen in Urin und Stuhl sowie Protoporphyrinämie gelten als Kennzeichen der Porphyrien, kommen aber häufiger im Kontext anderer Krankheiten vor, die die Hämbiosynthese nur sekundär betreffen. Bei diesen, klinisch symptomfreien, **sekundären Koproporphyrinurien**

sekundären Koproporphyrinurien

und Protoporphyrinämien handelt es sich entweder um metabolische Mitreaktionen des Porphyrinstoffwechsels bei einer heterogenen Gruppe von Krankheiten und Syndromen oder (insbesondere bei **Protoporphyrinämien**

Protoporphyrinämien)

um kompensatorische Reaktionen der Erythropoese bei erhöhtem Hämbedarf.

Dazu gehören toxische Leberschäden, Fettleber, Hepatitis, intra- und extrahepatische Cholestasen, Pankreatitis, Eisen- und Bilirubinstoffwechselstörungen (Dubin-Johnson‑, Rotor‑, Gilbert-Meulengracht- und Crigler-Najjar-Syndrom), HIV-Infektion, Hämolyse, neoplastische bzw. hämatologische Grunderkrankungen sowie Medikamentennebenwirkungen.

Durch Analysen von Porphyrinvorläufern, Porphyrinen und Enzymen in Urin, Stuhl, Plasma und Heparinblut können im Kontext klinischer Informationen und fachgerechter Interpretation sekundäre Porphyrinurien und Porphyrinämien von Porphyrien differenziert werden [13].

Bei der Bleivergiftung, Tyrosinämie Typ 1 und der ALADP sind die ALA- und weniger die PBG-Spiegel im Urin erhöht (Tab. 1; [14]).

#### Cave

Erhöhte Porphyrinausscheidungen in Urin und Stuhl sowie Protoporphyrinämie sind typische biochemische Zeichen der Porphyrien, kommen aber häufiger im Kontext anderer Krankheiten vor, die die Hämbiosynthese nur sekundär betreffen.

## Therapie

### Akute hepatische Porphyrien

Die Therapiemöglichkeiten bei akuten hepatischen Porphyrien sind in Tab. 4 zusammengefasst. Die regulatorische Therapie mit **Glucose**

Glucose

und Häm ist pathophysiologisch begründet. Die supprimierende Wirkung von Glucose auf die hepatische ALAS1 und damit auf die Hämsynthese wird über einen hepatischen Rezeptorkoaktivator vermittelt („peroxisome proliferator-activated receptor gamma coactivator 1‑alpha“, PGC-1α, [15]). Zeigen AHP-Patienten neurologische Ausfallerscheinungen, ist eine Hämtherapie indiziert. Bei frühzeitiger Gabe von **Hämarginat**

Hämarginat

kommt es meist innerhalb von 72 h zu einer Besserung [16]. Hämarginat kann allerdings zu **venösen Obliterationen**

venösen Obliterationen

führen. Alternativ werden deshalb Ports und Shunts angelegt. Die Gefahr, absehbar jeglichen venösen Zugang zu verlieren, und progrediente neurologische Symptome trotz adäquater Therapie sollten die Überprüfung und Verlaufsbeobachtung in einem Porphyriezentrum nach sich ziehen. Die **Lebertransplantation**

Lebertransplantation

als Ultima Ratio führt zu einer vollständigen sowie dauerhaften biochemischen und klinischen Remission [17]. Damit wird einerseits die Lokalisation dieser Stoffwechselstörungen in der Leber offenbar und andererseits die Indolenz der medullären Hämsynthese gegenüber diesen Enzymdefekten transparent. Durch die prophylaktische Gabe (sog. Intervalltherapie) von Hämarginat kann eine Verringerung klinischer Manifestationen erreicht werden. Die konsekutive Gabe in kurzen Zeitintervallen (< 3 Wochen) wird jedoch zunehmend kritisch bewertet. Im Hämarginat enthaltenes Eisen und toxisch-inflammatorische Nebenwirkungen können zum Wirkverlust führen.

Zur Behandlung der ovulozyklischen Form der akuten intermittierenden Porphyrie mit rekurrierenden prämenstruellen Manifestationen werden agonistische **LHRH-Analoga**

LHRH-Analoga

(LHRH: luteinisierendes Hormon-Releasing-Hormon) mit Erfolg angewandt. Im Tiermodell und in einer Klinische-Phase-1-Studie konnte gezeigt werden, dass die Inhibition der hepatischen *ALAS1*-m-RNA durch monatlich s.c. applizierte Doppelstrang-RNA-Fragmente (**Givosiran**

Givosiran)

therapeutisch effektiv ist. Die an Galaktose gekoppelten Doppelstrang-RNA-Fragmente werden über den **Asialoglykoproteinrezeptor**

Asialoglykoproteinrezeptor

in Hepatozyten aufgenommen und intrazellulär zu Einzelstrang-RNA gespalten (20 Basenpaare Länge), die selektiv an die komplementäre *ALAS1*-m-RNA binden. Es folgen eine Verminderung der Translation des ALAS1-Proteins und somit Reduzierung der ALAS1-Aktivitätskonzentration. In der ENVISION-Studie wurden 94 symptomatische Patienten mit akuter hepatischer Porphyrie aus 18 Ländern, prospektiv randomisiert, entweder mit Givosiran, 2,5 mg/kgKG, oder Placebo behandelt [18]. Bei Patienten mit AIP konnte unter Givosirananwendung (*n* = 46) gegenüber Placebo (*n* = 43) die auf 12 Monate hochgerechnete Häufigkeit von Attacken im Mittel um 74 % reduziert werden (primärer Endpunkt). In der Givosirangruppe konnten ein signifikanter Konzentrationsabfall von ALA, PBG, des täglichen Schmerz-Scores und ein verringerter Bedarf an Hämtherapie gezeigt werden (sekundäre Endpunkte). Im Vergleich zu Placebo wurden vermehrt Hautreaktion am Injektionsort, Exanthem, Übelkeit, Fatigue, renale Nebenwirkungen (7 vs. 15 %) und erhöhte Konzentrationen der Serumaminotransferasen (2 vs. 15 %) beobachtet. Die Behandlung wird insbesondere den Patienten mit AIP mit wiederkehrenden klinischen Manifestationen und chronischen Symptomen gerecht [19].

**Table 4:** 

**1. Absetzen porphyrinogener Medikamente**^**a**^** und intensivmedizinische Überwachung**
**2. Kalorien**: Infusionen 24 kcal/kgKG und Tag (Glucose 4 g/kgKG und Tag, Protein) *Cave:* Refeeding-Syndrom, zunehmende Hyponatriämie! Serumnatrium, -kalium, -magnesium,-phosphat mindestens täglich überwachen
**3. Hämarginat**, bei schwerem Verlauf und neurologischer Symptomatik 3 mg/kgKG und Tag (max. 250 mg/Tag) i.v., 30 min über großlumige Vene bzw. ZVK an bis zu 4 aufeinander folgenden Tagen *Cave:* Hämpräparate sind vasotoxisch und führen zu signifikanten venösen Obliterationen. Viele Zentren empfehlen, Hämpräparate in Humanalbumin (z. B. in 100 ml, 20 %ig) zu lösen. Nachspülen der Vene mit 150 ml physiologischer NaCl-Lösung
**4. Weitere Maßnahmen** Elektrolyt- und Volumenkontrolle bzw. -ausgleich: Bei Hyponatriämie (< 125 mmol/l): Infusion mit 150 ml einer hyperosmolaren Natriumchloridlösung (3 %ig) über 20 min. Nachfolgend Kontrolle der Serumnatriumkonzentration. Dieser Therapieschritt wird wiederholt, bis ein Anstieg der Serumnatriumkonzentration um 5 mmol/l erreicht ist. Die weitere Infusionstherapie mit isotonischer Natriumchloridlösung (0,9 %ig) unter initial stündlicher Überwachung der Serumnatriumkonzentration. Den Serumnatriumwert in den ersten 24 h nicht über 10 mmol/l erhöhen, in den folgenden Tagen nicht über 8 mmol/l pro Tag Bei Schmerzen Acetylsalicylsäure, Morphinderivate, Gabapentin Bei Tachykardie und Hypertonie Propranolol, Metoprolol, Valsartan Bei Unruhe oder Brechreiz Lorazepam, Chlorpromazin, Chloralhydrat, Ondansetron Bei Ileussymptomatik Neostigmin Bei Atemlähmung assistierte oder kontrollierte Beatmung (evtl. Tracheotomie) Bei Infektionen Penicilline, Cephalosporine, Imipenem, Gentamicin, Amikacin, Vancomycin Bei Paresen sofort physiotherapeutische Maßnahmen

*ZVK* zentraler Venenkatheter

^a^www.drugs-porphyria.org

Die Patienten sollten über Faktoren, die die Erkrankung auslösen, informiert werden (Medikamente, Fasten, Stress, Alkohol, Rauchen). **Internationale Porphyrieausweise**

Internationale Porphyrieausweise

werden über den Hersteller von Hämarginat, Recordati Rare Diseases Orphan Europe, kostenlos zur Verfügung gestellt [18], sollten jedoch nur von einem die Diagnose sichernden, erfahrenen Porphyriezentrum ausgestellt werden, um die Zementierung von Fehldiagnosen zu vermeiden. Prophylaktische Maßnahmen zur Lebensführung sind für den weiteren Verlauf einer AHP entscheidend. Mithilfe einer **kalorienreichen Ernährung**

kalorienreichen Ernährung

wird die Latenzphase stabilisiert. Die Patienten sollten ggf. Traubenzuckerstücke oder Dextro-Sirup mit sich führen, deren/dessen Einnahme bei beginnenden abdominellen Schmerzen eine kritische Symptomentwicklung verhindern kann.

#### Merke

- Treten neurologische Symptome hinzu ist eine Hämtherapie indiziert.
- Die Behandlung mit Givosiran erscheint insbesondere bei AHP-Patienten mit wiederkehrenden klinischen Manifestationen und unzureichendem Ansprechen auf Häm indiziert.

### Porphyria cutanea tarda

Hydroxychloroquintherapie und Eisendepletion via **Aderlass**

Aderlass

sind wirksame „First-line“-Maßnahmen bei Porphyria cutanea tarda [20, 21]. Hydroxychloroquin stimuliert die renale Exkretion von Porphyrinen, begleitet von einer Mobilisation aus den peripheren Geweben. Deshalb kommt es innerhalb der ersten 3 Monate zur weiteren Zunahme der Ausscheidung von Porphyrinen im Urin und möglicherweise auch der Lichtempfindlichkeit. Eine Behandlung mit Hydroxychloroquin in niedriger Dosierung gilt als Therapie der Wahl (2-mal/Woche 100 mg). Meist wird nach 3 Monaten eine klinische und nach 6 bis 12 Monaten auch eine biochemische Remission erreicht. Bei PCT und homozygoter Mutation C282Y muss mit Aderlässen wie bei Hämochromatose behandelt werden.

Bei PCT und ausschließlich heterozygoten Mutationen im HFE-Gen (keine Veränderungen in den *TFR2, Hämojuvelin*- sowie *Ferroportingenen*) ist die Behandlung mit Hydroxychloroquin ausreichend, da sich keine schwere Eisenüberladung entwickelt. Aderlass und Hydroxychloroquin können kombiniert werden. In einer prospektiven Studie zeigte die Therapie mit Chloroquin vs. Aderlass eine vergleichbare Effektivität. Im Vergleich zu Aderlass waren unter der pharmakologischen Therapie mit Chloroquin ein Jahr nach Therapie die Rezidive erhöht (20 % vs. 36 %). **Alkoholkarenz**

Alkoholkarenz

bewirkt bereits eine klinische Besserung mit metabolischem Rückgang der Porphyrinakkumulation. Frauen müssen hormonale Kontrazeptiva bzw. eine evtl. Hormonersatztherapie absetzen. Die hoch effektive antivirale Therapie der Hepatitis C führte ebenso zur klinischen und zur biochemischen Remission der PCT.

#### Merke

Hydroxychloroquintherapie, Eisendepletion und antivirale Therapie (bei Hepatitis C) sind wirksame „First-line“-Maßnahmen bei Porphyria cutanea tarda.

### Erythropoetische Protoporphyrie

Bei einer Protoporphyrie ist **adäquater Lichtschutz**

adäquater Lichtschutz

unabdingbar. Da eine Empfindlichkeit v. a. auch gegenüber sichtbarem (blauem) Licht besteht, sind herkömmliche Sonnenschutzcremes, die gegen UV-Strahlung (besonders UV-B) schützen, für gewöhnlich unzureichend. Reflektierender Sonnenschutz auf der Basis von Titandioxid oder Zinkoxid schützt sowohl gegen UV‑A, UV‑B und sichtbares Licht und ist deshalb oft wirkungsvoller. Mitunter helfen diese Präparate nicht oder sie sind unverträglich. Die Lichtempfindlichkeit impliziert die strikte Notwendigkeit einer **Vitamin-D-Überwachung**

Vitamin-D-Überwachung

und -Substitution. Das α‑MSH-Analogon Afamelanotid wird als kleiner Stift alle 3 Monate s.c. implantiert und ist zur Anwendung in Porphyriezentren zugelassen. Nicht wenige Betroffene können durch Afamelanotid erstmalig ein weitgehend unbeeinträchtigtes Leben mit Sonnenlichtexposition führen [22].

Bei ausgeprägter Cholestase und fibrotischem Umbau (Zirrhose) ist eine Lebertransplantation unausweichlich. Plasma- und Erythrozytapherese können toxisches PPIX effektiv entfernen und ggf. die Zeit bis zu einer Lebertransplantation überbrücken. Die erste erfolgreiche Stammzelltransplantation wurde in Schweden durchgeführt. Es gibt Hinweise, dass eine **Eisensubstitution**

Eisensubstitution

die Protoporphyrinkonzentrationen verringert und damit die klinischen Zeichen bei XLP dramatisch verbessert [23]. Dies ist pathophysiologisch erwartbar, da die Funktion der Ferrochelatase unbeeinträchtigt ist und das Enzym das kumulierte Protoporphyrin durch Chelatisierung von Eisen oder Zink verbraucht. Obwohl auch bei EPP ein „Eisenmangel“ beobachtet werden kann, kommt es bei einer Eisensubstitution zu einer direkten Induktion der Erythropoese über das geschwindigkeitsbestimmende Enzym (ALAS2) mit erhöhter Protoporphyrinbildung und korrespondierend zunehmender Lichtempfindlichkeit [24].

#### Merke

Patienten mit EPP und XLP können durch Afamelanotidanwendung erstmalig ein weitgehend unbeeinträchtigtes Leben mit Sonnenlicht führen.

#### Internetadressen

www.porphyrie.de

www.drugs-porphyria.org

www.hgmd.cf.ac.uk

www.porphyria-europe.com

## Fazit für die Praxis

- Porphyrien umfassen Stoffwechselkrankheiten der Hämbiosynthese, die durch spezifische biochemische Muster von Porphyrinen und Porphyrinvorläufern in Urin (10 ml Spontanurin sind für die Diagnostik völlig ausreichend), Stuhl und Blut diagnostiziert und differenziert werden.
- Bei unklaren abdominalen und/oder neuropsychiatrischen Symptomen sowie bei einer Fotodermatose sollten Porphyrien grundsätzlich differenzialdiagnostisch in Betracht gezogen werden.
- Die Hyponatriämie ist für akute Porphyrien ein wegweisender Befund. Der Nachweis von erhöhten Konzentrationen der Koproporphyrinen im Urin bei vielen Erkrankungen (sekundäre Koproporphyrinurie) führt häufig zur Fehldiagnose einer „Porphyrie“. Europäisch zertifizierte Porphyriezentren (porphyria.eu) sollten die Diagnose einer Porphyrie grundsätzlich bestätigen.
- Die Behandlungsmöglichkeiten für Porphyrien haben sich in letzter Zeit erheblich erweitert. Neue Therapien ermöglichen die Lichtexposition oder adressieren selektiv dysfunktionelle bzw. dysregulierte Schritte der hepatischen bzw. der erythropoetischen Hämsynthese.

## CME-Fragebogen

Wo findet die Hämsynthese überwiegend statt?

Milz

Leber

Nieren

Knochenmark und Leber

Darmwand

Welcher der folgenden Faktoren prädisponiert am ehesten für eine Porphyria cutanea tarda?

Eisen

Kreatinin

Nikotin

O_2_-Mangel

Zink

Mit welcher Infektion ist die Porphyria cutanea tarda häufig assoziiert?

Hepatitis-B-Virus

Hepatitis-C-Virus

Epstein-Barr-Virus

Humanes Papillomavirus Typ 6

Hantavirus

Eine 35-jährige Patientin kommt wegen einer akut aufgetretenen Rotfärbung ihres Urins in Ihre Sprechstunde. Außerdem bestehen seit einigen Tagen Bauchschmerzen und Kribbelparästhesien v. a. der Hände. Die Laborbefunde sind bis auf einen Natriumwert von 129 mmol/l unauffällig. Für welche Erkrankung sprechen diese Befunde am ehesten?

Erythropoetische Protoporphyrie

Erythropoetische Harder-Porphyrie

Porphyria cutanea tarda

Kongenitale erythropoetische Porphyrie

Akute intermittierende Porphyrie

Eine 43-jährige Patientin wird Ihnen mit dem Zufallsbefund einer vergrößerten Leber, die aufgrund von abdominellen Schmerzen aufgefallen war, zugewiesen. Im Rahmen der Laboruntersuchung zeigen sich erhöhte Konzentrationen von Bilirubin und der Cholestaseparameter. In der Sonographie ist eine Splenomegalie auffallend. In der Anamnese gibt die Patientin seit der frühen Kindheit eine ausgeprägte Lichtempfindlichkeit an. Es kommt zu Rötungen und quälenden Schmerzen an lichtexponierten Arealen. Für welche Erkrankung sprechen diese Befunde am ehesten?

Erythropoetische Protoporphyrie

Hämochromatose

Porphyria cutanea tarda

Doss-Porphyrie

Akute intermittierende Porphyrie

Welche medikamentöse Therapie kommt bei der Porphyria cutanea tarda zum Einsatz?

Azathioprin

Hydroxychloroquin

Prednisolon

Mycophenolsäure

Ciclosporin

Welche medikamentöse Therapie kommt bei der akuten intermittierenden Porphyrie zum Einsatz?

Givosiran

Metformin

Rosuvastatin

Ursodeoxycholsäure

Afamelanotid

Eine 21-jährige Patientin mit einer bekannten akuten intermittierenden Porphyrie wird mit Schmerzen in den Extremitäten, Halluzinationen, Tetraparese, respiratorischer Insuffizienz und rotem Urin auf eine Intensivstation übernommen. Welche Maßnahme ist eher *nicht* dringlich?

Porphyrinanalytik und i.v.-Therapie mit Hämarginat

Infusionen 24 kcal/kgKG und Tag (Glucose 4 g/kgKG und Tag, Protein)

Auswahl nichtporphyrinogener Medikamente

Überwachung der Elektrolyte (Natrium, Kalium, Magnesium, Phosphat)

Hepatitis-C-Testung

Ein 55-jähriger Patient mit bekanntem Diabetes Typ 2, Nikotinabusus und regelmäßigem Alkoholkonsum entwickelt erstmalig im Sommerurlaub Blasen, Erosionen und eine vermehrte Hautverletzlichkeit an beiden Handrücken. Im Serum sind Ferritin, Alaninaminotransferase (ALT) und Aspartataminotransferase (AST) um das 3‑, 2‑ bzw. 1,5-Fache oberhalb des Normwertes erhöht. In der Sonographie zeigt sich eine Fettleber mit fokal vermehrten echoreichen Rundherden. Welche Erkrankung kommt sehr wahrscheinlich in Betracht?

Sonnenlichtallergie

Neurodermitis

Porphyria cutanea tarda

Hämochromatose

Sarkoidose

Bei einer 32-jährigen Patientin mit schweren kolikartigen Bauchschmerzen wurde eine Choledocholithiasis gefunden und endoskopisch behandelt. In der Notaufnahme wurde initial wegen dunklem Urin eine Porphyriediagnostik veranlasst. Die Konzentrationen der Gesamtporphyrine und des Koproporphyrins waren im Spontanurin (20 ml, auf Kreatinin bezogen) auf das 1,5-Fache des oberen Normwertes erhöht, der Porphobilinogen(PBG)- und der 5‑Aminolävulinsäure(ALA)-Wert normal. Wie würden Sie reagieren?

Urin über 24 h sammeln und erneut analysieren

Urinbefunde in 4 Wochen kontrollieren

Eine Mutationsanalyse auf Vorliegen einer hereditären Koproporphyrie veranlassen.

Die Koproporphyrinisomere im Urin bestimmen.

Den Befund dieser typischen sekundären Porphyrinurie bei Cholestase nicht weiter verfolgen.

## Abkürzungen

ADH: antidiuretisches Hormon
AHP: Akute hepatische Porphyrie
AIP: Akute intermittierende Porphyrie
ALA: 5‑Aminolävulinsäure
ALAD: 5‑Aminolävulinsäure-Dehydratase
ALADP/DP: 5‑Aminolävulinsäure-Dehydratase-Defekt-Porphyrie/Doss-Porphyrie
ALAS: 5‑Aminolävulinsäure-Synthase
CAR: „Constitutive androstane receptor“
CEP: Kongenitale erythropoetische Porphyrie
CPOX: Koproporphyrinogenoxidase
CYP: Zytochrom P450
EPP: Erythropoetische Protoporphyrie
FECH: Ferrochelatase
GNPAT: Glycerolphosphat-O-Acyltransferase
HCP: Hereditäre Koproporphyrie
HEP: Hepatoerythropoetische Porphyrie
HFE: Hereditary Fe (Eisen)
HIV: „Human immunodeficiency virus“
LHRH: Luteinisierendes Hormon-Releasing-Hormon
OMIM: Online Mendelian Inheritance in Man
PBG: Porphobilinogen
PBGD: Porphobilinogendesaminase
PCT: Porphyria cutanea tarda
PGC-1α: „Peroxisome proliferator-activated receptor gamma coactivator 1‑alpha“
PPIX: Protoporphyrin IX
PXR: Pregnan-X-Rezeptor
UROD: Uroporphyrinogendecarboxylase
UROS: Uroporphyrinogen-III(Ko)-Synthase
VP: Porphyria variegata
XLP: X‑chromosomale Protoporphyrie
